# A Delphi study to build consensus on the definition and use of big data in obesity research

**DOI:** 10.1038/s41366-018-0313-9

**Published:** 2019-01-17

**Authors:** Christina Vogel, Stephen Zwolinsky, Claire Griffiths, Matthew Hobbs, Emily Henderson, Emma Wilkins

**Affiliations:** 1 MRC Lifecourse Epidemiology Unit, University of Southampton, SO16 6YD Southampton, UK; 2 School of Sport, Leeds Beckett University, LS6 3QQ Leeds, UK; 3 GeoHealth Laboratory, Geospatial Research Institute, University of Canterbury, Christchurch, New Zealand; 4 Institute of Health & Society, Newcastle University, Newcastle-upon-Tyne, NE1 4LP UK

**Keywords:** Risk factors, Epidemiology

## Abstract

**Background:**

‘Big data’ has great potential to help address the global health challenge of obesity. However, lack of clarity with regard to the definition of big data and frameworks for effectively using big data in the context of obesity research may be hindering progress. The aim of this study was to establish agreed approaches for the use of big data in obesity-related research.

**Methods:**

A Delphi method of consensus development was used, comprising three survey rounds. In Round 1, participants were asked to rate agreement/disagreement with 77 statements across seven domains relating to definitions of, and approaches to, using big data in the context of obesity research. Participants were also asked to contribute further ideas in relation to these topics, which were incorporated as new statements (*n* = 8) in Round 2. In Rounds 2 and 3 participants re-appraised their ratings in view of the group consensus.

**Results:**

Ninety-six experts active in obesity-related research were invited to participate. Of these, 36/96 completed Round 1 (37.5% response rate), 29/36 completed Round 2 (80.6% response rate) and 26/29 completed Round 3 (89.7% response rate). Consensus (defined as > 70% agreement) was achieved for 90.6% (*n* = 77) of statements, with 100% consensus achieved for the Definition of Big Data, Data Governance, and Quality and Inference domains.

**Conclusions:**

Experts agreed that big data was more nuanced than the oft-cited definition of ‘volume, variety and velocity’, and includes quantitative, qualitative, observational or intervention data from a range of sources that have been collected for research or other purposes. Experts repeatedly called for third party action, for example to develop frameworks for reporting and ethics, to clarify data governance requirements, to support training and skill development and to facilitate sharing of big data. Further advocacy will be required to encourage organisations to adopt these roles.

## Introduction

Obesity is a persistent public health problem that no country has successfully addressed [1]. Novel datasets, particularly those not initially collected for obesity research, could provide important information to improve understanding of the interaction between, and relative influence of, the various determinants of obesity. Sources of continuously collected data have grown rapidly in recent years as a result of digitalised systems, and significant improvements in data processing and storage capabilities [2–4]. These large data sources are sometimes called ‘big data’. The first two papers [5, 6] in this series demonstrate the increasing attention big data is garnering for obesity research and the wide variety of commercial and government data sources that are available and fit for purpose. They highlight the great potential big data has for formulating and evaluating policy, developing intervention initiatives for obesity prevention, and understanding its multiple determinants and their interactions. Nevertheless, big data in obesity and population research remains underutilised [7].

The slow adoption of big data in global efforts to reduce obesity prevalence may, in part, stem from a lack of clarity about the exact meaning of the term and what it entails for obesity-related research. Definitions can help describe the work needed and provide directions about associated skill, resource and infrastructure requirements [8]. There is no single, agreed definition of big data, yet it is often typified as being extensive in volume, derived from a wide variety of sources or collected at great velocity [2–4]. In the context of obesity research, the term big data often refers to novel data sets that have been collected for purposes other than health research, which may provide added value to more traditional data sources [3]. However, it has been debated whether traditional datasets, such as administratively collected medical records or large cohort studies, can also be deemed big data, particularly if they are linked to more novel data sources [4, 7, 9]. Reaching agreement on what big data encompasses in the context of obesity will help to increase precision and understanding of the term by researchers, and aid future activity in this field.

A clear definition is one that captures the meaning, use and function of a particular topic or concept, and guides researchers to develop a cohesive body of empirical evidence [10]. Clear definitions are valuable when developing research questions and presenting study findings because interpretations of loosely defined terms will be shaped by perceptions of the audience, who commonly have different educational, professional and cultural experiences [11]. Imprecise definitions can make it difficult to agree on what is being researched and may lead to studies examining disparate or heterogeneous concepts that can hinder development and collation of the evidence base. For example, there have been several recent funding calls related to the use of big data in public health research, including obesity [12–14]. One project, ‘Big O’, that was awarded funding under the Horizon 2020  Big Data funding call uses mobile phones to purposively collect data on obesity-related behaviours such as food intake [15]. While these data were deemed to meet the definition of big data in this instance, purposively collected data is a grey area, which is not always considered to constitute big data [5]. A definition of what constitutes big data would provide clear guidance and reduce inefficiencies for funders and researchers in understanding which proposed projects meet the funding criteria. It could also facilitate the use of particular datasets, and similar exposure and outcome variables which are imperative to enable meta-analyses and systematic reviews to summarise scientific evidence [16].

Developing a clear definition of big data for obesity research could also aid consistency and transparency across contributors from different industries and settings. One potential pitfall to progress in this field is the management and sharing of data [3, 4]. The General Data Protection Regulation (GDPR) [17] recently introduced across Europe provides a clear example of where not having a definition of big data for obesity research could be problematic. These new data regulations are accompanied by the threat of fines of up to 10 million Euros or 2% of global turnover for any personal data breaches including those related to collecting, processing or sharing data [18]. For egregious breaches, fines of 20 million Euro or 4% of global turnover are proposed. These substantial fines offer significant reason to hinder data collectors, particularly commercial companies, from sharing their data with researchers despite the potential for public benefit. Exemptions to the data regulations do exist for purposes in the *public interest* or for *research*. However, these terms have not been explicitly defined and appropriate safeguards relating to storage, processing and sharing are still required to protect anonymity [17]. Having a clear definition of big data for obesity research that could be adopted by member states may help to specify cases where exemptions to the regulations are appropriate.

Previous literature regarding big data has focused on analysis techniques or terminology [3, 9]. It has largely overlooked the practical elements necessary to guide successful acquisition and appropriate utilisation of big data for non-communicable conditions such as obesity [7, 19]. Thus in addition to a clear definition, there is need for an architecture for utilising big data in obesity research. This will help facilitate consistent and effective approaches and help overcome any issues academics may encounter. Previous authors exploring the usage of big data in health and social care have highlighted a number of challenges including data acquisition restrictions or costs that limit universal accessibility of datasets [4, 7]. Ethical and legal questions also exist around ownership and access, such as whether commercial data should be made available to research institutions for potential societal benefit [20]. Further, adherence to ethical principles and data protection regulations is problematic when individuals have not explicitly consented for their data to be used or linked to other data [21]. Additional challenges also include the need for data management and analysis skills that lie outside traditional public health training [22]. Furthermore, there are questions around data governance and reporting requirements as increasing numbers of people become involved in data creation and collation [23]. Similar concerns persist regarding bias, which can be introduced through poor data or study design quality, and in turn limit the ability to draw casual inference [24].

In recognition of the issues surrounding the use of big data, the Economic and Social Research Council (ESRC) funded a Strategic Network for Obesity (Obesity Network) [25]. This network is a collaboration of 40 members from academia, industry, health charities and the public sector that explored emerging forms of data to catalyse an approach to obesity at five network meetings between 2015 and 2017. These meetings highlighted that challenges to the effective application of big data to obesity research are experienced similarly across obesity-related disciplines by members of the Organisation for Economic Co-operation and Development (OECD). While there may be some minor differences in their use to account for local or cultural issues, the acquisition and employment of big data should be transferable between countries to enable international comparison. Thus the aim of the present study was to establish an agreed approach for using big data in obesity-related research in OECD countries. A Delphi survey design was used to integrate international and interdisciplinary perspectives from academics with expertise in obesity research and/or experience applying big data to examine obesity, dietary or physical activity outcomes.

The objectives of this study were to build consensus among international experts in the field of obesity on: (i) a definition of big data that is appropriate for obesity research; and (ii) consistent and effective approaches academic researchers can take to use big data to address obesity with particular consideration of the issues relating to: Data Acquisition, Ethics, Governance, Training and Infrastructure, Reporting and Transparency, and Quality and Inference.

## Methods

### Study design

The Delphi technique has proven to be a reliable measurement instrument in developing new concepts and setting the direction of future-orientated research [26]. The technique seeks the opinion of a group of experts in order to assess the extent of agreement and to resolve disagreement on an issue [27]. It has been used to establish consensus across a range of subject areas, with several in the field of obesity and obesity-related behaviours [28–31].

**Fig. 1:**
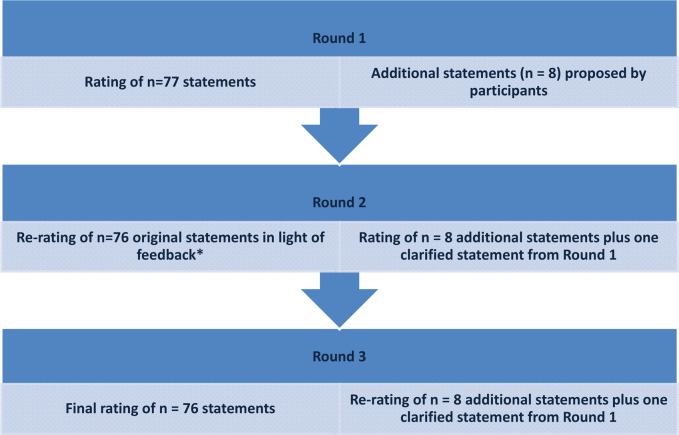
Flow diagram illustrating the three survey rounds of the Delphi study. *One statement that appeared in Round 1 was removed, and a new clarified version was added in Round 2

The Delphi process comprised three rounds (Fig. 1). In Round 1, participants were asked to independently rank a total of 77 statements, across seven domains, using a 4-point Likert scale (’strongly agree’, ‘agree’, ‘disagree’, ‘strongly disagree’). It has been demonstrated that 4-point scales produce stable findings in Delphi studies [32]. For each statement, participants were given the option to select ‘don’t know’ as an alternative response. This option was added because big data is an emerging and challenging field, and feedback from pilot testing indicated that some participants may not know how to answer certain statements. Furthermore, this enabled identification of domains that are particularly unclear and require additional attention. A free-text response was available to participants within each of the survey domains, providing the opportunity to elaborate or explain responses. In Round 1, data on participant demographics were also collected including: gender, year of birth, country of residence, current job position, highest educational qualification obtained and time (in years) working in the field of obesity research.

In Round 2, each participant received an individualised survey comprising 85 statements, across seven domains. This survey included 76 statements from Round 1, which were presented alongside participants’ own responses from Round 1, as well as the group’s collective response (percentage agreement/disagreement) to each statement. All ‘don’t know’ responses were excluded from the group response. Participants were asked to reconsider their responses in light of the group’s responses. Round 2 also included eight new statements derived from the free-text responses to Round 1. Further, the free-text responses from Round 1 helped to clarify one statement which was then added as a new statement in Round 2. There was no option for free-text responses in Round 2.

In Round 3, each participant received an individualised survey, comprising all 85 statements from Round 2 presented alongside the participants’ own responses and the group’s response (percentage agreement/disagreement) from Round 2. Participants were asked to reconsider their responses in light of the group’s responses for a final time.

Three survey rounds were employed because this enables adequate reflection on group responses and is considered optimal to reach consensus [33]. Three survey rounds also allowed free-text responses from Round 1 to be incorporated as new statements in Round 2 and re-evaluated in light of the group consensus in Round 3 (Fig. 1). A third survey round for these new statements was not required because consensus was achieved on all except one statement which had response split that meant consensus would be unlikely. All surveys were administered using Qualtrics (Provo, USA), and survey links were distributed via email.

### Survey development

Statements for the survey were developed from study team’s expertise, intelligence from the Obesity Network and a review of the literature [5]. To meet the study objectives, the survey was divided into two sections. The first included statements to establish a definition of big data and the second, sought consensus on approaches to using big data in obesity research. Statement development capitalised on an existing survey carried out as part of Obesity Network activities. Members’ responses to the question: ‘what is big data?’ were used to create statements for the first section of the survey; and the second section used members’ responses to the questions: ‘what are your concerns with using big data for research?’ and ‘what are the main challenges within your work in terms of big data?’ Four authors (SZ, CG, MH and EW) independently analysed the responses to identify themes and propose statements. These statements were supplemented and refined in light of the literature review findings and knowledge of the research team.

A total of 14 statements were included in the first section of the survey. The second section included 63 statements across six domains: Data Acquisition, Ethics, Governance, Training and Infrastructure, Reporting and Transparency, and Quality and Inference. These domains have also been identified as important considerations surrounding the use of big data in health and social care [7, 20–24]. The survey statements were constructed to highlight the key challenges and opportunities relating to each domain, and to agree effective approaches to address these challenges.

The survey was piloted with seven academics who had a range of obesity-related experience, including professors in statistical epidemiology, nutritional science and behavioural science. An iterative processes of feedback was undertaken to improve the structure and readability of statements, and to determine whether any additional statements were needed.

### Expert panel recruitment

In Delphi exercises, a minimum of 12 respondents is generally considered to be sufficient to enable consensus to be achieved, larger sample sizes can provide diminishing returns regarding the validity of the findings [34–39]. Nevertheless, Delphi sample sizes depend more on group dynamics in reaching consensus than their statistical power [40, 41]. A non-probability purposive sample of ninety-six participants were invited via email to participate in this Delphi survey. Sampling was purposive to ensure that invited participants met the inclusion criteria. All participants were required to be 18 years or above, fluent English speakers, actively conducting research in obesity or obesity-related fields, and affiliated with an academic institution from an OECD country. The invited participants were either members of the Obesity Network (*n* = 34), academics known to members of the Obesity Network (*n* = 45), or authors of published articles relating to obesity and big data identified from the first paper in this series [5] (*n* = 17). To complete the Delphi process, participants were required to respond across all three rounds. Therefore, those who did not respond to Round 2 were not invited to participate in Round 3. A dropout rate of 20% was expected over the three rounds, in accordance with previous Delphi studies [32, 42]. This study aimed to recruit and complete the process with 30 experts.

### Ethics

Ethical approval for this study was granted by the Local Research Ethics Committee at Leeds Beckett University. All participants provided informed consent to take part at the beginning of the process as part of the online survey. All data were handled in accordance with UK data protection regulations.

### Data analysis

Descriptive statistics were used to describe participants’ demographic characteristics and group responses to each statement in all three rounds. Consensus was defined as > 70% of participants agreeing/strongly agreeing or disagreeing/strongly disagreeing with a statement in Round 3. This level of agreement has been considered appropriate in previous Delphi studies [40, 42, 43]. All ‘don’t know’ responses were excluded from the group response to ensure that the reported percentage agreement or disagreement for each statement represented the consensus among only those who felt they knew the answer. Stability of consensus was considered reached if the between round group responses varied by ≤10% [44]. Analyses were conducted using SPSS for windows version 24 [45].

## Results

Of the 96 experts invited to participate in this Delphi study, 36 participants completed Round 1 (37.5% response rate), 29 of 36 completed Round 2 (80.6% response rate) and 26 of 29 completed Round 3 (89.7% response rate). Table 1 presents the demographic characteristics of participants in each round. Gender distribution was consistent across the three rounds, with only a slightly higher percentage of males. Participants’ mean age ranged from 42 to 44 years across the three rounds, and approximately three quarters resided in the UK. The majority of respondents were senior academics, had doctoral degrees and had been working in the field of obesity research for ≥ 5 years.

**Table 1:** Demographic characteristics of Delphi participants

	Round 1 (*n* = 36)	Round 2 (*n* = 29)	Round 3 (*n* = 26)
Gender			
Male	52.8%	55.2%	53.8%
Female	47.2%	44.8%	46.2%
Mean age in years (SD)	43.9 (10.9)	42.3 (9.8)	41.5 (9.7)
Country of residence			
UK	72.2%	75.9%	80.8%
USA	11.1%	6.9%	7.7%
Netherlands	8.3%	10.3%	7.7%
New Zealand	2.8%	3.4%	3.8%
Australia	2.8%	3.4%	0.0%
Ireland	2.8%	0.0%	0.0%
Current role			
Professor	30.6%	27.6%	26.9%
Associate Professor	13.9%	13.8%	15.4%
Lecturer	16.7%	17.2%	15.4%
Research Fellow	27.8%	31.0%	30.8%
PhD student	5.6%	6.9%	7.7%
Other	5.6%	3.4%	3.8%
Education			
Doctoral Degree	91.7%	93.1%	92.3%
Professional Fellow	2.8%	0.0%	0.0%
Master’s Degree	5.6%	6.9%	7.7%
Years working in the field			
10+ years	52.8%	44.8%	46.2%
6–9 years	14.0%	17.2%	15.3%
4–5 years	19.4%	20.7%	19.2%
1–3 years	13.9%	17.2%	19.2%

Table 2 shows a summary of the Delphi statements for each of the seven domains. The number of statements where consensus was achieved improved for each domain from Round 1 to Round 3. In Round 1, consensus was achieved for 64.5% (*n* = 49) of the 76 statements. In Round 2, consensus was achieved for 81.2% (*n* = 69) of the 85 statements and this rose to 90.6% (*n* = 77) in Round 3. There was variation in the proportion of statements that achieved consensus between domains but the proportion of consensus increased in each subsequent round across all domains. By Round 3, 100% consensus was achieved for three domains (Definition of Big Data (*n* = 15), Data Governance (*n* = 5), and Quality and Inference (*n* = 11); the lowest level of consensus was 75.0% for Training and Infrastructure (*n* = 9). Stability of consensus (<10% variation) was achieved between Round 2 and Round 3 for four of the seven domains.

**Table 2:** Summary of grouped statements by domain

Statement domains	Number of statements in each domain			Proportion of statements where consensus was achieved (*n*)		
	Round 1	Round 2	Round 3	Round 1	Round 2	Round 3
Definition of Big Data	14	15	15	64.3% (9)	80.0% (12)	100.0% (15)
Data Acquisition	13^a^	16	16	38.5% (5)	68.8% (11)	81.3% (13)
Ethics	13	15	15	61.5% (8)	80.0% (12)	93.3% (14)
Data Governance^b^	5^a^	5	5	80.0% (4)	100.0% (5)	100.0% (5)
Training and Infrastructure^b^	11	12	12	63.6% (7)	75.0% (9)	75.0% (9)
Reporting and Transparency^b^	9	11	11	77.8% (7)	90.9% (10)	90.9% (10)
Quality and Inference^b^	11	11	11	81.8% (9)	90.9% (10)	100.0% (11)
Totals	76	85	85	64.5% (49)	81.2% (69)	90.6% (77)

Note: Consensus was achieved when 70% of participants strongly agreed/agreed or strongly disagreed/disagreed with a statement

^a^Statements in this round of this domain include responses where ‘don’t know’ exceeded 30% of total responses

^b^Stability of consensus (<10% variation) was achieved between Round 2 and Round 3

Table 3 presents the group responses to each survey statement included in the definition of Big Data domain. By Round 3, consensus was achieved for all 15 statements, with 80% (*n* = 12) of these statements achieving consensus in Round 2. Three statements needed three rounds before consensus was reached. Table 4 shows the group responses to the Delphi statements as they appeared in the participant survey across the six domains. The Delphi survey sought to identify agreed approaches to using big data in obesity research. For the Data Acquisition domain, eleven (68.8%) of the 16 statements reached consensus in Round 2; by Round 3 consensus has been achieved on 13 statements (81.3%). Three statements did not reach consensus and these related to participant knowledge, big data owners’ responsibilities for promoting their data, and data protection regulations. For the Ethics domain, 14 (93.3%) of the 15 statements reached consensus by Round 3, up from 12 (80.0%) in Round 2. One statement relating to the ethics of commercial companies withholding big datasets could not be agreed upon by the group. Consensus was achieved for all five (100%) statements included in the Data Governance domain in Rounds 2 and 3. In Round 1, however, more than 30% of participants reported not knowing whether data governance processes were clear for data owners and controllers. For the Training and Infrastructure domain, consensus was reached for 9 (75.0%) of the 12 statements in Rounds 2 and 3. No consensus was achieved for three statements, highlighting differences in time, training and equipment needs for big data analyses across researchers and institutions. One statement out of 11 in the Reporting and Transparency domain could not be agreed by the expert panel; this statement described the need to report costs associated with acquiring big data. The remaining 10 (90.9%) statements achieved consensus in Round 2 and Round 3. Consensus was attained for all 11 (100.0%) statements included in the Quality and Inference domain by Round 3, an improvement from 9 (81.8%) in Round 1 and 10 (90.9%) in Round 2. Across all domains, the direction of agreement for statements not reaching consensus until the third round did not change from the earlier rounds, it only strengthened.

**Table 3:** Responses to statements included in the Definition of Big Data domain

Big Data….	Round 1 (*n* = 36)		Round 2 (*n* = 29)		Round 3 (*n* = 26)	
	Agree %	Disagree %	Agree %	Disagree %	Agree %	Disagree %
1. Always has a large sample size	**77.8%**	22.2%	**75.9%**	24.1%	**88.5%**	11.5%
2. Always requires additional computing power	68.6%	31.4%	69.0%	31.0%	**80.8%**	19.2%
3. Is never collected for research purposes (i.e. there is no a priori research question)	25.7%	**74.3%**	21.4%	**78.6%**	15.4%	**84.6%**
4. Is always observational	40.0%	60.0%	22.2%	**77.8%**	23.1%	**76.9%**
5. Does not require specialist mathematical or data science analytical skills	12.1%	**87.9%**	7.1%	**92.9%**	8.0%	**92.0%**
6. Does not require specialist knowledge of database management	15.6%	**84.4%**	14.8%	**85.2%**	12.5%	**87.5%**
7. Does not require knowledge of computer programming	42.4%	57.6%	37.0%	63.0%	25.0%	**75.0%**
8. Is always digital	61.8%	38.2%	66.7%	33.3%	**72.0%**	28.0%
9. Does not include qualitative data	35.3%	64.7%	17.9%	**82.1%**	23.1%	**76.9%**
10. Includes government data sets	**94.3%**	5.7%	**96.6%**	3.4%	**96.2%**	3.8%
11. Includes cohort data sets	**86.1%**	13.9%	**92.9%**	7.1%	**96.2%**	3.8%
12. Includes commercial data sets	**97.2%**	2.8%	**96.6%**	3.4%	**96.2%**	3.8%
13. Includes routine data sets	**94.4%**	5.6%	**96.6%**	3.4%	**100.0%**	0.0%
14. Always includes more than one data set	16.7%	**83.3%**	17.2%	**82.8%**	15.4%	**84.6%**
15. Big data always has at least one of: • large volume (e.g. in terms of sample size, number of variables or measurement occasions), • variety (e.g. in terms of the types of variable), or • velocity (e.g. is generated at speed)	–	–	**93.1%**	6.9%	**92.3%**	7.7%

Note: Bold % denotes that 70% consensus was achieved

**Table 4:** Responses to statements included in the six domains which sought agreed approaches to using big data in obesity research

	Round 1 (*n* = 36)		Round 2 (*n* = 29)		Round 3 (*n* = 26)	
	Agree %	Disagree %	Agree %	Disagree %	Agree %	Disagree %
*Data Acquisition*						
1. There is not equal access to big datasets for all academic researchers	**97.1%**	2.9%	**96.6%**	3.4%	**100.0%**	0.0%
2. There is not equal access to big datasets across academic institutions or non-academic researchers	**97.1%**	3.0%	**96.6%**	3.4%	**100.0%**	0.0%
3. I don’t know what big data are available to use for research purposes	58.3%	41.7%	**75.9%**	24.1%	**76.9%**	23.1%
4. I don’t know how to access big data for research purposes	47.2%	52.8%	48.3%	51.7%	57.7%	42.3%
5. Accessing big data for research purposes takes too long	**75.0%**	25.0%	**95.5%**	4.5%	**95.2%**	4.8%
6. Timescales for access to big data limit their utility for obesity research	55.2%	44.8%	**72.0%**	28.0%	**73.9%**	26.1%
7. Negotiating access to big data for obesity research is a challenge	**94.1%**	5.9%	**96.6%**	3.4%	**96.2%**	3.8%
8. Access to big data should be provided via a third party centre/organisation that is independent both from the data owner and the researcher	**76.0%**^**a**^	24.0%^a^	**83.3%**	16.7%	**82.6%**	17.4%
9. Third party organisations (i.e. those outside of a university) should be responsible for promoting the awareness of big data for use in obesity research	46.2%	53.8%	20.8%	**79.2%**	25.0%	**75.0%**
10. It is the responsibility of data owners to make their data available	65.7%	34.3%	69.0%	31.0%	**73.1%**	26.9%
11. Data owners are responsible for making others aware of the availability of their data	48.5%	51.5%	35.7%	64.3%	36.0%	64.0%
12. It is the responsibility of individual research institutions to identify and negotiate access to big data sources	56.7%	43.3%	63.0%	37.0%	**75.0%**	25.0%
13. The cost attached to the use of big data is a major barrier to its use	62.1%	37.9%	**79.2%**	20.8%	**81.0%**	19.0%
14. Data protection regulations unduly restrict the use of big data in obesity research	–	**–**	50.0%	50.0%	42.1%	57.9%
15. Government legislation is needed to encourage commercial organisations to share their data for obesity research	–	–	**80.8%**	19.2%	**84.0%**	16.0%
16. Big data should be made available via third party organisations who should be responsible for protecting both commercially sensitive and individually sensitive data	–	–	**83.3%**	16.7%	**87.0%**	13.0%
*Ethics*						
1. It is unethical to use big data in obesity research when consent has not been obtained for this purpose	12.9%	**87.1%**	11.1%	**88.9%**	7.7%	**92.3%**
2. Consent is a major ethical challenge for big data in obesity research	**77.4%**	22.6%	**85.2%**	14.8%	**84.0%**	16.0%
3. Big data from commercial sources is a potential conflict of interest	64.7%	35.3%	**78.6%**	21.4%	**80.8%**	19.2%
4. Ethical processes need reviewing in light of using big data in obesity research	**94.3%**	5.7%	**96.6%**	3.4%	**96.2%**	3.8%
5. Ethical processes unduly restrict the use of big data for obesity research	46.4%	53.6%	36.4%	63.6%	30.0%	**70.0%**
6. There are high confidentially risks when using big data for obesity research	38.2%	61.8%	26.9%	**73.1%**	20.8%	**79.2%**
7. It is the responsibility of individual research institutions to ensure that big data is used ethically	**94.4%**	5.6%	**100.0%**	0.0%	**100.0%**	0.0%
8. It is the responsibility of individual researchers to ensure that big data is used ethically	**97.2%**	2.8%	**100.0%**	0.0%	**100.0%**	0.0%
9. It is the responsibility of data owners to ensure that big data is used ethically	**94.4%**	5.6%	**93.1%**	6.9%	**92.3%**	7.7%
10. It is unethical of commercial companies to withhold big data sets that could be used to identify determinants of obesity and opportunities for intervention	48.5%	51.5%	39.9%	60.7%	38.5%	61.5%
11. Using big data for obesity research doesn’t cause harm because no further contact with individuals or communities is made	58.6%	41.4%	**73.9%**	26.1%	**76.2%**	23.8%
12. An ethical framework is required to review big data research proposals through formal research processes	**93.9%**	6.1%	**93.1%**	6.9%	**96.2%**	3.8%
13. An ethical framework should be developed by independent bodies with no conflicts of interest	**79.4%**	20.6%	**86.2%**	13.8%	**92.3%**	7.7%
14. Ethical processes should distinguish between open data already in the public domain and secondary data not already in the public domain, which may contain both commercially and individually sensitive data	–	–	**92.9%**	7.1%	**96.0%**	4.0%
15. It is unethical NOT to use big data where it is available, even when informed consent has not been provided, if it will help address obesity	–	–	30.4%	69.6%	14.3%	**85.7%**
*Data Governance*						
1. The data governance requirements associated with using big data in obesity research are clear	17.2%	**82.8%**	16.0%	**84.0%**	16.7%	**83.3%**
2. Data governance processes are clear for data controllers	34.8%^a^	65.2%^a^	13.6%	**86.4%**	15.0%	**85.0%**
3. Data governance processes are clear for researchers	25.8%	**74.2%**	12.0%	**88.0%**	12.0%	**88.0%**
4. Data governance processes are clear for data owners	20.8%^a^	**79.2%**^a^	13.6%	**86.4%**	15.8%	**84.2%**
5. Ownership of big data can be ambiguous (e.g. for wearables/activity tracking technology the owner could be taken to be the organisation who collates/manages the data, or the individual people the data relates to)	**94.3%**	5.7%	**96.6%**	3.4%	**96.2%**	3.8%
*Training and Infrastructure*						
1. Big data requires novel/non-traditional analysis techniques	**80.0%**	20.0%	**92.9%**	7.1%	**96.0%**	4.0%
2. Researchers need specialist training to link big data	**85.3%**	14.7%	**92.9%**	7.1%	**92.0%**	8.0%
3. Researchers need specialist training to manage big data	**88.6%**	11.4%	**89.3%**	10.7%	**92.0%**	8.0%
4. Researchers need specialist training to analyse big data	**83.3%**	16.7%	**89.7%**	10.3%	**88.5%**	11.5%
5. There is insufficient training available to me, regarding the handling of big data and analysis	59.4%	40.6%	61.5%	38.5%	59.1%	40.9%
6. The cost of training courses in big data analysis techniques prevents me from using these datasets	23.3%	**76.7%**	19.2%	**80.8%**	17.4%	**82.6%**
7. My institution has limited equipment/systems necessary for handling big data (i.e. computer memory, secure networked systems etc.)	41.9%	58.1%	37.0%	63.0%	37.5%	62.5%
8. It is the responsibility of individual universities to improve their training and infrastructure to use big data in obesity research	**80.6%**	19.4%	**93.1%**	6.9%	**88.5%**	11.5%
9. It is the responsibility of professional organisations, including funding organisations, to provide more training around big data	**82.9%**	17.1%	**86.2%**	13.8%	**88.5%**	11.5%
10. The time involved in preparing big datasets for analysis prevents me from using these datasets	40.0%	60.0%	48.3%	51.7%	48.0%	52.0%
11. There are no training or infrastructure issues that prevent me from using big data for obesity research	41.2%	58.8%	25.9%	**74.1%**	20.8%	**79.2%**
12. Collaboration that draws on varied skill sets is needed to appropriately handle big data in obesity research	–	–	**93.1%**	6.9%	**92.3%**	7.7%
*Reporting and Transparency*						
1. The provenance (source and date of collection) of big data is adequately reported in peer-reviewed literature	25.0%	**75.0%**	12.5%	**87.5%**	4.2%	**95.8%**
2. The methods originally used to collect big data are adequately reported in peer-reviewed literature	29.4%	**70.6%**	7.1%	**92.9%**	7.7%	**92.3%**
3. Procedures used to clean and process (e.g. re-code) big data are adequately reported in peer-reviewed literature	8.6%	**91.4%**	7.1%	**92.9%**	8.0%	**92.0%**
4. The content of big data sources are adequately reported in peer-reviewed literature	20.6%	**79.4%**	7.4%	**92.6%**	12.0%	**88.0%**
5. The processes used to link big data sources (e.g. geocoding techniques) are adequately reported in peer-reviewed literature	19.4%	**80.6%**	11.1%	**88.9%**	8.3%	**91.7%**
6. Inadequate reporting of big data and associated methods in peer-reviewed literature means study findings cannot be usefully interpreted	65.7%	34.3%	**78.6%**	21.4%	**84.6%**	15.4%
7. The costs associated with obtaining big data should be reported in peer-reviewed literature	51.6%	48.4%	51.9%	48.1%	62.5%	37.5%
8. To improve big data related obesity research, standardised reporting frameworks are required	**84.8%**	15.2%	**89.3%**	10.7%	**92.3%**	7.7%
9. Academic journals have a responsibility to enforce the use of reporting frameworks for big data	**82.9%**	17.1%	**86.2%**	13.8%	**92.3%**	7.7%
10. Where contractual restrictions exist around the reporting of data, these should be noted when disseminating research findings	–	–	**100.0%**	0.0%	**100.0%**	0.0%
11. Reporting needs to be independent of the data owner to reduce potential conflicts of interest	–	–	**72.0%**	28.0%	**79.2%**	20.8%
*Quality and Inference*						
1. Big data from commercial organisations results in an increased risk of bias	58.8%	41.2%	**73.1%**	26.9%	**80.0%**	20.0%
2. Standardised quality checks of the data [i.e. how data was collected, missing data] are required from the data provider	**91.4%**	8.6%	**89.3%**	10.7%	**96.2%**	3.8%
3. Big data should be used irrespective of quality in obesity research	19.4%	**80.6%**	13.8%	**86.2%**	11.5%	**88.5%**
4. It is important to acknowledge methodological limitations of big data used in obesity research	**100.0%**	0.0%	**93.1%**	6.9%	**100.0%**	0.0%
5. Statistically significant results need to be interpreted with caution when using big datasets in obesity research	**91.2%**	8.8%	**96.4%**	3.6%	**96.0%**	4.0%
6. Outputs from research using big data are rarely misinterpreted	11.1%	**88.9%**	8.3%	**91.7%**	9.1%	**90.9%**
7. There is an over reliance on big data in obesity research despite its potential bias	17.2%	**82.8%**	12.0%	**88.0%**	16.7%	**83.3%**
8. The emergence of big data has negatively impacted the use of traditional data sources	20.0%	**80.0%**	14.3%	**85.7%**	16.7%	**83.3%**
9. Big data is having an unhealthy steer on the obesity-related research agenda	13.8%	**86.2%**	14.3%	**85.7%**	15.4%	**84.6%**
10. Researchers have a responsibility to ensure that their results are correctly interpreted in view of any limitations	**100.0%**	0.0%	**100.0%**	0.0%	**100.0%**	0.0%
11. Big data obesity research should always consider inequalities in health or health behaviours as a measure of quality	57.6%	42.4%	69.2%	30.8%	**73.9%**	26.1%

Note: Bold % denotes that 70% consensus was achieved

^a^Proportion of ‘don’t know’ responses to this statement exceeded 30%

The proportion of participants that reported ‘don’t know’ to each statement in Round 3 is presented in Table S1, Supplementary material. The Definition of Big Data domain had the lowest proportion of ‘don’t know’ responses (1.5%), and the Data Governance domain had the highest (12.3%). None of the statements in Round 3 had ‘don’t know’ responses that exceeded 30% of the total responses.

## Discussion

This Delphi survey achieved consensus, from a panel of 26 international experts who completed three rounds, on 100% of the 15 statements proposed to develop a definition of big data for obesity research. Additionally, the survey reached consensus on 88.6% of statements put forward to describe approaches for researchers to effectively use big data in obesity-related studies. Descriptions of the panel agreement against the two aims of this study are outlined below under the subheadings ‘defining big data’ and ‘consistent approaches to using big data’.

### Defining big data

One definition that represents the consensus among the expert group on the full list of definition-specific descriptors is provided in the box below. This type of definition is likely to be important when communicating what big data is to those not familiar with the term or when ascertaining the circumstances in which big data applies to, or is exempt from, regulations. For audiences more familiar with big data, a shorter, more succinct definition may be more appropriate.

*Big data is always digital, has a large sample size, and a large volume or variety or velocity of variables that require additional computing power. It can include quantitative, qualitative, observational or interventional data from a wide range of sources (e.g. government, commercial, cohorts) that have been collected for research or other purposes, and may include one or several datasets. Specialist skills in computer programming, database management and data science analytics are usually required to analyse big data*.

This definition of big data determined by Delphi method draws upon the increasingly recognised definition of the three V’s of big data: volume, variety and velocity [2–4]. However, it provides greater detail about types of information equated with the term and the sources from which it can be acquired. It also recognises that training and computing resources required for big data extend beyond those traditionally used in obesity studies. This definition is consistent with descriptions provided in commentaries by authors from North America with regard to big data use in epidemiological or public health research [7, 24], providing confidence in the representativeness of our findings. The high level of agreement from this study’s expert group in how we define big data for obesity research supports the notion that big data’s defining characteristics are applicable across countries and in different research contexts.

Given the evolving nature of big data in obesity research in many countries, the key descriptors agreed upon can be employed in versatile ways. For example, peer-reviewed journals could require authors to follow a reporting protocol, such as BEE-COAST [6], when describing their big data studies and may define such studies using one or more of the definition descriptors agreed upon by the expert panel. These may include data type (e.g. requiring data to be digital with a large sample size, volume, variety and/or velocity) and source descriptors (e.g. requiring data to be from government, commercial or cohort sources) but could exclude the training descriptors.

### Consistent approaches to using big data

**Fig. 2:**
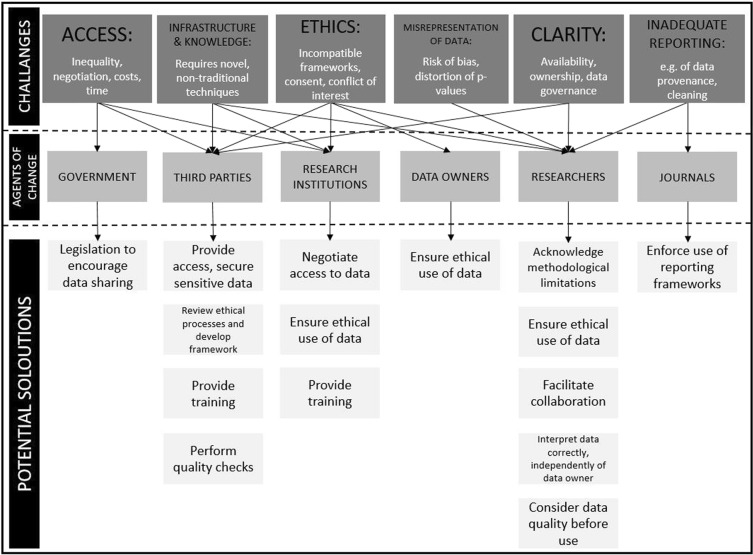
Challenges, solutions and agents of change for effective use of big data in obesity research

The consensus-building technique employed in this study identified a number of approaches that need to be consistently implemented by various stakeholders to optimise use of big data in obesity research. Figure 2 summarises six challenges the expert panel collectively identified as currently hindering effective use of big data and the recommended six different stakeholders groups who are optimally placed to become agents of change to overcome these challenges. Informed by the consensus achieved on 62 statements, the figure also illustrates the potential solutions the expert panel agreed could be enacted by each stakeholder group to facilitate effective and consistent use of big data in obesity-related research.

The results of this study identified a number of issues surrounding big data that have been previously noted, including disparities in acquisition due to cost, access and time constraints [4, 7], and ethical concerns regarding individual and commercial privacy and consent [3, 20, 23]. This Delphi study, however, expanded on previous literature by identifying practical actions to overcome these challenges. A recurring theme was around the need for third party action. For example, the experts agreed that there was a need for organisations that act independently of both data owners and researchers, to provide repositories of big datasets from various sources and ensure the protection of both individual identities and commercial sensitivities. A small number of such organisations already exist, including the Consumer Data Research Centre (CDRC) and Administrative Data Research Centre (ADRC) that form part of the ESRC-funded Administrative Data Research Network and are hosted by UK universtities [46, 47]. These centres provide access to a variety of data sources for the research community, potentially reducing time and financial costs.

Such third parties work with commercial and government organisations to encourage them to open up their data to researchers to help address societal issues like obesity. These repository centres could extend their current remit to include advocating for government legislation to require commercial organisations to share their data for obesity research as supported by the expert panel in this study. Such third party organisations can also safeguard commercially and individually sensitive data, by providing secure facilities for approved researchers to access linked, de-identified data. Techniques such as data perturbation can and are being used by third party repository centres to enable functionally anonymised data to be used in research [22].

The expert panel from this study also agreed that an ethical framework and data governance protocols need to be developed by a non-conflicted, independent body to guide appropriate use of big data by all stakeholders. Potential organisations could include the Obesity Network or internationally recognised professional organisations such as the World Obesity Federation or International Society for Behavioural Nutrition and Physical Activity. The results of this study provide useful information to draft ethical and governance protocols that could then be debated and subsequently agreed at international conferences. Specifying the requirements for different actors, including data owners, data controllers and researchers, will aid adherence to high ethical and data governance standards. Endorsement of the protocols by a range of professional and government organisations would facilitate their uptake and implementation by academic ethical committees, third party data repositories, researchers and data owners.

It has become increasingly recognised that the growth of big data requires specific analytic skills that are not traditionally incorporated into professional training courses for a number of sectors including public health and epidemiology [2, 9, 22, 48]. When considering training needs, panellists from this study recommended that universities, professional organisations and funding bodies provide more teaching in linking, managing and analysing big data. Such training opportunities could take the form of continuing professional development activities or incorporated into undergraduate and postgraduate curriculums. For example, professional organisations such as the World Obesity Federation could introduce training opportunities in machine learning techniques [7, 9, 49] as part of their E-learning modules. Data repository centres, including CDRC and ADRC mentioned above, also offer a range of short courses about big data linkage, management and analysis that are currently available to researchers to improve their confidence and skills in this area. While a number of online training facilities are freely available, funding bodies may need to do more to support skill development in big data analytics for researchers at all career stages.

The application of machine learning techniques to big data in obesity research has been shown to provide robust methods for handling missing and incorrectly recorded data eliminating the need to curate longitudinal datasets for analyses [50]. However, concerns about data quality and causal inference with big data have been acknowledged [7, 21] and were supported by the expert panel in this study. Big data sources may not be representative, and similar data sources may not reveal consistent results. Additionally, while larger sample sizes reduce the likelihood of random error, measurement error can introduce bias independent of a dataset’s sample size [3, 51]. The experts participating in this study agreed that the methodological limitations of big data, including selection bias, measurement error and risk of confounding, should always be acknowledged. They indicated the need for standardised reporting frameworks to improve transparency regarding data quality and facilitate appropriate inference. The BEE-COAST framework [6] has been shown to suitably summarise the important features of a number of big data sources. If this framework were to be enforced by academic journals, and details outlining the background to data collection, data ownership, content and the temporality of the dataset routinely described, concerns about conflicts of interest and data quality are likely to be systematically reduced. The third parties proposed above could take an active role in promoting the adoption of this framework by editorial boards of peer-reviewed journals in a similar way to which reporting frameworks for observational studies and systematic reviews have been embraced [52, 53].

### Strengths and weaknesses

This Delphi study gathered consensus on a range of topics relevant to the burgeoning global field of big data in academic obesity research, the findings for which have enabled the research team to develop initial guidance and areas for policy and research development. Drawing on an international network of obesity researchers funded to develop this field, views were gathered from a wide range of related disciplines. The size and composition of the expert panel may not be representative of all OECD countries and may therefore reduce the generalizability of the results. Nevertheless, one of the strengths of this paper is that the final sample size was more than double the lower limit threshold of 12 [39]. Given the global scale upon which this field operates, the Delphi consensus technique, which can be conducted online, was the appropriate tool for bringing together these views. In addition to identifying areas of consensus, the study was able to highlight areas where there is less certainty in the field, potentially requiring further exploration and a widening of disciplines to resolve these issues. While a strength of the study was its ability to access a network of colleagues in the field of obesity research, the authors of this study are members of the Obesity Network and this may have introduced some response bias. The response rates for each round of the study were 37.5%, 80.6% and 89.7% for Round 1, Round 2 and Round 3, respectively. Based on guidance from the NIHR Health Technology Assessment for this technique [39], we anticipated a dropout rate of 20% over the three rounds of consensus development. A main limitation of this study is that it does not offer definitive guidance; however, this study recommends independent parties draw upon these findings and others to create resources to improve consistency and quality of big data use in the field of obesity.

## Conclusion

With an expert panel, this study was able to reach consensus on the majority of statements included in this study. It was felt that the definition of big data in the context of obesity research was more nuanced than the simple and oft-cited three V’s of big data: ‘volume, variety and velocity’, and includes quantitative, qualitative, observational or interventional data from a wide range of sources (e.g. government, commercial, cohorts) that have been collected for research or other purposes. This definition can help position future discussions and frameworks around the use of big data in obesity research.

Experts identified a number of challenges that need to be resolved in order to more effectively use big data in obesity research. A recurring theme was the need for third party action, for example to develop frameworks for reporting and ethics, to clarify data governance requirements and to support training and skill development. The findings also indicate that third parties should play a role in arbitrating access to big data in order to protect commercial and individual confidentiality, as well as enable more equitable access to data and potentially reduce the time and financial costs to individual researchers and institutions. While organisations that fulfil some of these roles already exist, further advocacy will likely be needed to encourage organisations to adopt wider responsibilities. Individual researchers, research institutions and data owners also hold important roles in facilitating effective and ethical use of big data. Determining the responsibilities of different actors, and monitoring adherence to these responsibilities will not be simple, and may require government involvement.

## Supplementary information

Table S1
