# Supplementary material for: A Delphi study to build consensus on the definition and use of big data in obesity research
Source: Int J Obes (Lond). 2019 Jan 17;43(12):2573–86. doi: 10.1038/s41366-018-0313-9 (PMC6892733; doi:10.1038/s41366-018-0313-9)
Supplement: Supplementary file 1 — Table S1 [file 41366_2018_313_MOESM1_ESM.docx]

Table S1: Proportion of ‘Don’t Know’ responses by domain for survey statements in Round 3

| **STATEMENT NUMBER** | **Definition of**  **Big data** | **Data**  **Acquisition** | **Ethics** | **Data Governance** | **Training and Infrastructure** | **Reporting and Transparency** | **Quality and Inference** |
| --- | --- | --- | --- | --- | --- | --- | --- |
|  | **% (n)** | | | | | | |
| *1* | 0.0% (0) | 0.0% (0) | 0.0% (0) | 7.7% (2) | 3.8% (1) | 7.7% (2) | 3.8% (1) |
| *2* | 0.0% (0) | 0.0% (0) | 3.8% (1) | 23.1% (6) | 3.8% (1) | 0.0% (0) | 0.0% (0) |
| *3* | 0.0% (0) | 0.0% (0) | 0.0% (0) | 3.8% (1) | 3.8% (1) | 3.8% (1) | 0.0% (0) |
| *4* | 0.0% (0) | 0.0% (0) | 0.0% (0) | 26.9% (7) | 0.0% (0) | 3.8% (1) | 0.0% (0) |
| *5* | 3.8% (1) | 19.2% (5) | 23.1% (6) | 0.0% (0) | 15.4% (4) | 7.7% (2) | 3.8% (1) |
| *6* | 7.7% (2) | 11.5% (3) | 7.7% (2) | - | 11.5% (3) | 0.0% (0) | 15.4% (4) |
| *7* | 7.7% (2) | 0.0% (0) | 0.0% (0) | - | 7.7% (2) | 7.7% (2) | 7.7% (2) |
| *8* | 3.8% (1) | 11.5% (3) | 0.0% (0) | - | 0.0% (0) | 0.0% (0) | 7.7% (2) |
| *9* | 0.0% (0) | 7.7% (2) | 0.0% (0) | - | 0.0% (0) | 0.0% (0) | 0.0% (0) |
| *10* | 0.0% (0) | 0.0% (0) | 0.0% (0) | - | 3.8% (1) | 0.0% (0) | 0.0% (0) |
| *11* | 0.0% (0) | 3.8% (1) | 19.2% (5) | - | 7.7% (2) | 7.7% (2) | 11.5% (3) |
| *12* | 0.0% (0) | 7.7% (2) | 0.0% (0) | - | 0.0% (0) | - | - |
| *13* | 0.0% (0) | 19.2% (5) | 0.0% (0) | - | - | - | - |
| *14* | 0.0% (0) | 26.9% (7) | 3.8% (1) | - | - | - | - |
| *15* | 0.0% (0) | 3.8% (1) | 19.2% (5) | - | - | - | - |
| *16* | - | 11.5% (3) | - | - | - | - | - |
| **Domain total** | 1.5% (6) | 7.7% (32) | 5.1% (20) | 12.3% (16) | 4.8% (15) | 3.5% (10) | 4.5% (13) |

Note: Respondents n=26 in Round 3; the domain total is a product of the actual number of ‘don’t know’ responses by the possible number of ‘don’t know’ responses.
